# A complex case of *Nocardia* keratitis: challenges in diagnosis and therapy

**DOI:** 10.1590/S1678-9946202567019

**Published:** 2025-03-17

**Authors:** Maria Astrid Claudia, Ismi Zuhria

**Affiliations:** 1 Universitas Airlangga, Faculty of Medicine, Department of Ophthalmology, Surabaya, East Java, Indonesia; 2 Dr. Soetomo General Academic Hospital, Department of Ophthalmology, Surabaya, East Java, Indonesia

**Keywords:** Nocardia infection, Keratitis, Corneal diseases, Corneal trauma, Ulcerative keratitis

## Abstract

*Nocardia* keratitis is a rare cause of microbial keratitis, primarily affecting patients in tropical and subtropical regions. Its diagnostic challenge arises from this keratitis uncommon presentation, which often mimics other infectious keratitis types, leading to delays in appropriate treatment. This case report aims to elucidate the complexities of diagnosing and managing *Nocardia* keratitis of a 41-year-old male who had a chronic, progressively worsening wreath-pattern corneal infiltrate and hypopyon following ocular trauma. Initial empirical treatments were ineffective. Diagnostic confirmation via corneal scraping culture enabled targeted antimicrobial therapy. Subconjunctival amikacin and topical tobramycin led to gradual improvement, though complications such as corneal scarring and neovascularization remained, indicating potential need for surgical intervention. This case emphasizes the importance of high clinical suspicion and precise laboratory diagnostics in managing rare cases of *Nocardia* keratitis. Establishing standardized treatment guidelines for rare ocular infections could improve clinical outcomes.

## INTRODUCTION

*Nocardia* keratitis is an uncommon and often elusive cause of corneal infection, particularly rare on a global scale, with a prevalence below 2%^1^. This condition, though primarily found in tropical and subtropical regions, represents a significant diagnostic challenge for clinicians worldwide due to its atypical presentation and the scarcity of cases^2^. Initially misclassified as a fungal pathogen, *Nocardia* spp. is now recognized as a Gram-positive, branching filamentous bacterium with a unique morphological profile that can be misinterpreted under microscopic examination. The rarity and misclassification risks associated with *Nocardia* contribute to a substantial rate of delayed or erroneous diagnoses^3^.

Clinically, *Nocardia* keratitis often shows nonspecific symptoms, such as conjunctival injection, ocular pain, and photophobia, which resemble more common infectious keratitis causes like fungi or atypical mycobacteria^4^. This similarity in presentation frequently leads to empirical treatments for the pathogens that are more common, resulting in ineffective interventions and progression of the keratitis. Furthermore, the corneal "wreath-like" infiltrate characteristic with satellite lesions, while pathognomonic, is not consistently present, which complicates clinical diagnosis^5^. Treatment of *Nocardia* keratitis is similarly challenging, as standardized protocols lack due to the infection rarity^6^. Although amikacin has demonstrated efficacy as a first-line treatment, some *Nocardia* strains are resistant, necessitating alternative therapeutic strategies and possibly systemic treatment in severe or non-responsive cases. The slow progression of *Nocardia* infections, alongside the pathogen resistance profile and potential for poor visual outcomes, underscores the importance of accurate and timely diagnosis^7^.

This case report illustrates the complexity of diagnosing and managing *Nocardia* keratitis, aiming to enhance clinical awareness of its distinguishing features and therapeutic challenges, particularly in atypical or resistant presentations^8^.

The authors have obtained written informed consent from the patient for publication of this case report and accompanying images.

## CASE REPORT

A 41-year-old male presented with a four-week history of redness, pain, tearing, photophobia, and impaired vision in his left eye (LE), which was developed following an accidental ocular injury caused by a grasshopper while riding a motorcycle. The symptoms began approximately two weeks after the injury, initially mild but progressively worsening over time. He reported no symptoms in the right eye (RE) and no significant medical history, including the absence of systemic disease or prior eye surgery.

On initial visit to the ophthalmology clinic, his primary complaint was a gradually intensifying whitish appearance over the LE cornea, which failed to resolve with prescribed treatments, including natamycin and levofloxacin eye drops. Despite adherence to the treatment regimen, symptoms persisted and worsened, prompting further investigation.

**Figure 1:**
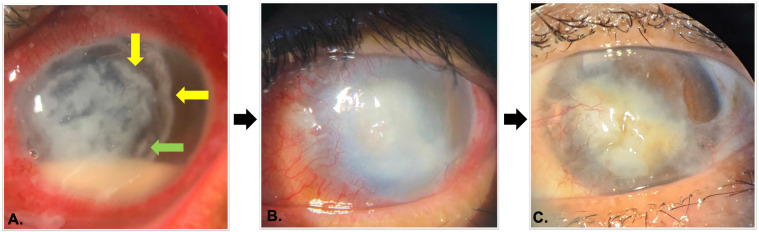
Slit lamp photographs of the LE: (A) Initial presentation: corneal "wreath-pattern" infiltrate (yellow arrows) with satellite lesions (green arrow) and hypopyon; (B) One month: After taking subconjunctival amikacin for five days, tobramycin ED, and cotrimoxazole tablets, hypopyon resolved completely, but peripheral corneal neovascularization emerged; (C) Six months after admission: resolved *Nocardia* keratitis showed leucoma and neovascularization, the surrounding cornea remains clear.

Clinical examination revealed visual acuity of 5/5 in the RE and only hand motion in the LE, with normal intraocular pressure in both eyes on palpation. The anterior segment examination of the RE was unremarkable. However, the LE showed significant findings, including conjunctival and limbal injection, mucopurulent discharge, and a pathognomonic corneal infiltrate with a unique "wreath-pattern" arrangement, accompanied by a satellite lesion in the mid-periphery. This infiltrate measured approximately 10 × 8 mm, with a positive fluorescein staining test indicating an epithelial defect. No feathery edge infiltrates were observed. A hypopyon measuring 2 mm in height was observed in the anterior chamber, however, neither cells nor flare were detected. Further examination of the iris, pupil, and lens was challenging due to the dense infiltrate. B-scan ultrasonography confirmed no abnormalities in the posterior segment, ruling out further intraocular involvement. Figure 1A shows the slit lamp examination of the left eye upon initial presentation, demonstrating a corneal ‘wreath-pattern’ infiltrate (yellow arrows) with satellite lesions (green arrow) and a visible hypopyon. Microbiological evaluation of corneal scrapings, including KOH, Gram staining, and culture, was performed. Direct microscopic examination with KOH showed no evidence of fungal elements, whereas Gram staining revealed Gram-positive filamentous organisms.

Initial empirical therapy included intravenous ceftriaxone twice per day, hourly moxifloxacin eye drops for the LE, ketoconazole twice per day, and doxycycline tablets twice a day. Despite the aggressive treatment, the patient's condition showed minimal improvement, with hypopyon height increasing after initial intervention, necessitating paracentesis. Following the procedure, the hypopyon was temporarily resolved but recurred within two days, measuring 0.5 mm in height.

**Figure 2:**
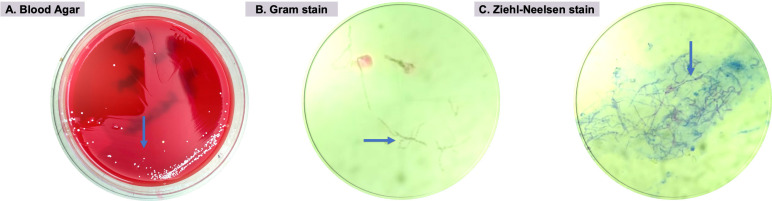
Microbiological examinations: (A) Blood agar inoculated with corneal scrapings showing white, dry, and chalky colonies (blue arrow); (B) Gram-positive thin branching filaments bacteria (blue arrow) on Gram stain; (C) Positive acid-fast staining (blue arrow).

After a seven-day incubation period, microbiological analysis of the corneal scrapings revealed the growth of *Nocardia* species, which was characterized as a rare Gram-positive branching filamentous bacterium that showed marked susceptibility to both amikacin and tobramycin. Gram staining demonstrated the presence of Gram-positive, thin branching filamentous bacteria, which also tested positive for acid-fast staining. Such characteristics were consistent with *Nocardia* species, with no evidence of fungal growth being observed. The treatment was then adjusted to include subconjunctival amikacin injections daily and tobramycin eye drops every two hours for the LE, aligned with the antibiotic sensitivity results. Figure 2A shows the blood agar culture with characteristic colonies (blue arrow), Figure 2B demonstrates the Gram-positive branching filaments, and Figure 2C shows the positive acid-fast staining.

Following ten days of hospitalization, the patient was discharged showing reduced symptoms and a smaller infiltrate area. Subsequent evaluations revealed a reduction in both the infiltrate and epithelial defect size, however, hypopyon persisted. In response, Cotrimoxazole was added to the oral treatment plan at a dosage of 960 mg twice a day. One month after discharge, the patient's infiltrate had reduced further, measuring around 5 × 6 mm, with a fluorescein-positive area of 2 × 2 mm centrally, and complete resolution of hypopyon. Figure 1B shows the corneal infiltrate reduced in size with complete resolution of the hypopyon. Neovascularization was observed along the corneal periphery, a sequela commonly associated with prolonged infection.

At six months follow-up, corneal leucoma and neovascularization were observed, with no epithelial defect noted on fluorescein staining. Although visual prognosis was limited due to the scar formation, the infection had been effectively managed, and further surgical intervention, including possible keratoplasty, was considered for visual rehabilitation. Figure 1C captures the final corneal appearance with resolved infection, leucoma, and peripheral neovascularization.

## DISCUSSION

*Nocardia* keratitis is a rare and complex ocular infection, posing diagnostic and therapeutic challenges for clinicians due to its unusual presentation and limited awareness^1^. This case highlights the diagnostic hurdles, treatment strategies, and implications for managing such infections effectively. The diagnosis of *Nocardia* keratitis is often delayed due to its nonspecific presentation and rarity. Literature suggests *Nocardia* accounts for less than 2% of microbial keratitis globally, with higher prevalence rates in tropical regions^9^. Clinically, the condition often mimics fungal keratitis, leading to initial empirical antifungal treatment. In our patient, the delayed diagnosis and initial empirical antifungal therapy illustrate this common diagnostic pitfall, as fungal pathogens are often suspected first when patients show atypical corneal infiltrates^4^. Underdiagnosis can lead to delayed appropriate treatment, increasing the risk of complications such as corneal melting, neovascularization, and ultimately, visual impairment^10^.

Classic clinical features of *Nocardia* keratitis, such as the "wreath-pattern" infiltrate and satellite lesions, are useful diagnostic clues but may not always be present^5^. Therefore, clinicians must maintain a high level of suspicion when dealing with keratitis that does not respond to conventional therapy. In this case, a definitive diagnosis was established via microscopic examination of culture isolates, which demonstrated Gram-positive branching filamentous organisms that were also positive on acid-fast staining, characteristic of *Nocardia* species. The utilization of specific staining techniques, including Gram and Ziehl-Neelsen stains, enabled differentiation of *Nocardia* spp. from similar pathogens, thereby preventing underdiagnosis. The organism showed partial acid-fast properties with Ziehl-Neelsen staining and underwent complete decolorization with 20% sulfuric acid, a characteristic that facilitated differentiation from *Mycobacterial* species^1,9^. While 10% potassium hydroxide preparation is conventionally used for fungal detection, the microscopic examination demonstrated very fine, intertwined, branching filamentous structures characteristic of *Nocardia* species, distinctly different from the broader septate hyphal elements or yeast forms typically seen in fungal keratitis^9,11^. Managing *Nocardia* keratitis is equally challenging, largely due to the absence of standardized treatment protocols and the pathogen's variable susceptibility profile^1^. Literature and case series suggest *Nocardia* is typically sensitive to amikacin and tobramycin, which were utilized effectively in this case^2^. In our case, after *Nocardia* was confirmed via culture and sensitivity results, we administered subconjunctival amikacin and topical tobramycin, which led to a gradual but incomplete resolution of the corneal infiltrate.

Challenges in selecting an appropriate antibiotic regimen persist due to the variability in *Nocardia's* susceptibility patterns. Limited data exist on antibiotic resistance specific to ocular *Nocardia* infections, although resistance to aminoglycosides has been observed in other cases^12^. The frequent reliance on empiric antifungal therapy when *Nocardia* is suspected but not confirmed illustrates a critical area for improvement, as early, targeted antimicrobial therapy can significantly reduce the risk of complications and antibiotic resistance^10^.

*Nocardia* keratitis can lead to severe complications, impacting long-term visual outcomes^2^. In this case, despite microbial resolution, the patient's residual leucoma and corneal neovascularization necessitate further surgical intervention, such as keratoplasty, to improve vision^13^. The literature suggests while surgical intervention is relatively rare, it may be essential in cases with poor initial response to medical therapy or severe corneal thinning^14^.

This case emphasizes the need for clinician awareness and a high index of suspicion when treating atypical microbial keratitis. As *Nocardia* remains a rare but clinically significant pathogen, ophthalmologists should include it in the differential diagnosis for cases that are unresponsive to standard therapy^2^. Early microbiological workup, including advanced diagnostic techniques, is essential for accurate identification, which can significantly influence treatment outcomes^9^.

This case contributes to valuable insights into the presentation, diagnosis, and management of *Nocardia* keratitis. By highlighting a comprehensive clinical course and documenting specific imaging and laboratory findings, it serves as an informative guide for clinicians encountering similar cases. Recognizing pathognomonic features, such as the "wreath-pattern" infiltrate, can aid early diagnosis and targeted antimicrobial therapy guided by sensitivity testing is crucial for effective management^9^.

Future research is needed to explore antibiotic resistance patterns and to establish evidence-based guidelines for treating *Nocardia* infections^1^. Such research could provide more definitive recommendations, particularly concerning therapy duration, follow-up protocols, and indications for surgical intervention. By advancing our understanding of this rare condition, clinicians will be better equipped to manage its unique challenges, ultimately improving patient outcomes in *Nocardia* keratitis cases^12^.

## CONCLUSION

*Nocardia* keratitis shows unique diagnostic and therapeutic challenges due to its rarity, indolent progression, and misidentification potential. This case highlights the importance of early recognition of pathognomonic features and appropriate laboratory diagnostics to prevent delays in treatment. Despite microbial resolution, complications such as corneal scarring and neovascularization emphasize the need for prolonged follow-up and, at times, surgical intervention. Standardized treatment protocols for such rare infections could improve patient outcomes. This report aims to enhance clinical awareness of *Nocardia* keratitis, underscoring the value of targeted, evidence-based management in atypical ocular infections.
